# Remote Augmented Reality Versus Traditional Simulation for Team Leader Assessment in a Cardiac Arrest Scenario: Noninferiority Randomized Controlled Trial

**DOI:** 10.2196/84367

**Published:** 2026-03-16

**Authors:** Renan Gianotto-Oliveira, Marcos Rojas, Maria Queiroz, Flávia Zanchetta, Anabel Ferrari, Lucas Kojima, Alexandre Paula, Bruno Campos, Dario Cecilio-Fernandes, Thomas J Caruso

**Affiliations:** 1 School of Medical Sciences, University of Campinas, Campinas, Brazil; 2 Department of Anesthesiology, Perioperative, and Pain Medicine, Stanford School of Medicine, 453 Quarry Road, office 422A, Palo Alto, CA, 94305, United States, 1 (650) 723-2300; 3 Erasmus MC University Medical Centre Rotterdam, Institute of Medical Education Research Rotterdam, Rotterdam, The Netherlands

**Keywords:** augmented reality, AR, simulation-based education, advanced cardiac life support, resuscitation, digital health education, randomized controlled trial

## Abstract

**Background:**

Simulation-based education is crucial for training health care professionals in advanced cardiac life support. However, access to high-fidelity in-person simulation is frequently limited by geographic, logistical, and financial constraints. Augmented reality (AR) offers the potential to deliver remote, immersive training experiences that may overcome these barriers, but its effectiveness compared with traditional simulation remains uncertain.

**Objective:**

This study aimed to determine whether remote AR simulation is noninferior to traditional in-person simulation for assessing team leader performance during a ventricular fibrillation cardiac arrest scenario.

**Methods:**

This noninferiority randomized trial enrolled participants at the State University of Campinas (UNICAMP), Brazil, and used cross-continental remote instruction from Stanford University (in the United States) for the AR arm. A total of 50 health care professionals were randomized to either remote AR simulation with a geographically distant instructor (n=25) or traditional in-person simulation (n=25). All participants completed an identical ventricular fibrillation cardiac arrest case as team leaders. Leader performance was assessed using an adapted, validated checklist-based instrument for cognitive leadership and an observational behavioral measure (Behaviorally Anchored Rating Scale). Secondary outcomes included AR participants’ evaluations of usability and ergonomics.

**Results:**

A total of 42 participants fully completed the study procedures (remote AR group: n=22; traditional in-person group: n=20). The AR group demonstrated noninferior performance compared to the traditional group across all outcomes. The mean checklist scores were 41.6 (SD 6.2) and 42.6 (SD 5.8) in the remote AR group and traditional in-person group, respectively. The AR group’s 95% CI (38.9‐44.4) was above the 20% noninferiority threshold of 34.1. Usability and ergonomics were favorably reported by most participants.

**Conclusions:**

Participants in the remote AR simulation demonstrated noninferior team leader decision-making and behavioral performance compared with those in traditional in-person simulation. These findings suggest that remote AR may be a viable strategy to expand access to scenario-based assessment of cardiac arrest leadership, particularly in resource-limited settings. AR participants also reported high usability and low ergonomic burden, indicating comfortable headset use.

## Introduction

In an advanced cardiac life support (ACLS), the performance of the team leader is a critical determinant of resuscitation quality and patient outcomes. Effective leadership requires rapid clinical decision-making, clear communication, and coordinated task delegation to optimize team performance under pressure [1]. Deficiencies in leadership behaviors can compromise adherence to resuscitation algorithms and reduce overall team efficiency, even when individual team members possess strong technical skills [2,3]. Simulation-based assessments provide structured environments to evaluate these competencies objectively, integrating both technical and nontechnical skills essential for safe patient care [4,5].

Despite its educational value, traditional in-person simulation faces persistent barriers. High costs, logistical complexity, and the requirement for physical facilities and expert facilitators limit its scalability, particularly in resource-constrained or geographically dispersed settings [6-8]. These challenges highlight the need for accessible strategies capable of delivering high-fidelity assessment without the infrastructure demands of traditional simulation.

Immersive technologies offer innovative pathways to mitigate these limitations. Augmented reality (AR) simulation has gained traction in health professions education as a way to deliver interactive scenarios for learners. In contrast to traditional virtual reality (VR), which replaces the surrounding environment with a fully virtual setting, AR augments the real world with digital information while keeping learners embedded in a shared physical space. Newer VR headsets now use a video “pass through” mode, replacing fully computer-generated imagery in VR with the natural world, simulating an AR experience. This distinction matters for clinical training that depends on direct interaction with teammates, natural face-to-face communication, and the manipulation of authentic equipment and task trainers, features that can be harder to preserve when the environment is fully virtual. By overlaying interactive digital elements onto real-world settings through head-mounted displays (HMDs), AR enables learners to engage in realistic, team-based scenarios while preserving face-to-face communication and allowing real-time remote facilitation [9]. This ability to blend virtual overlays with task trainers and clinical equipment enables mixed reality experiences that can mirror key features of conventional simulation. Despite growing adoption, existing studies have less often examined AR through an instructional design lens or clearly demonstrated what educational benefit AR provides beyond other simulation approaches [10,11]. AR preserves the essential elements of conventional simulation, such as realism, interactivity, and teamwork, while providing a practical alternative to in-person assessments.

Because this modality requires minimal equipment and setup time, AR can be implemented flexibly in multipurpose clinical spaces, reducing scheduling constraints that often restrict access to dedicated simulation centers. Prior studies suggest that it enhances learner engagement and reproduces essential elements of pediatric advanced life support and ACLS, including leadership under pressure and team coordination [12]. Participants report high usability, and AR simulations can support complex task execution [13,14].

Remote instruction and assessment through AR simulations offers promising potential for providing standardized evaluation of clinical performance in settings where traditional simulation is costly, difficult to scale, or inaccessible. To address this gap, we conducted an international, randomized noninferiority trial evaluating team leader performance in a ventricular fibrillation (VF) cardiac arrest scenario. The primary outcome was to determine whether AR-based assessment yielded noninferior leadership performance compared with traditional in-person simulation. Secondary outcomes measured the AR participants’ evaluation of usability and ergonomics.

## Methods

### Design

This international trial used a parallel, noninferiority design with a 1:1 allocation ratio to compare team leader performance in a simulated VF cardiac arrest scenario. All participants were recruited and completed study procedures at the State University of Campinas (UNICAMP), Brazil. Stanford University functioned as the remote instructor site for the AR arm. Participants were randomized to complete either a remote instructor–led AR simulation or a traditional in-person simulation. Recruitment occurred between June 20, 2024, and August 2, 2024, across 2 academic centers. The study was prospectively registered at ClinicalTrials.gov (NCT06326450; March 15, 2024).

Following trial commencement, no significant modifications were made to the study protocol, including eligibility criteria or protocol design, ensuring uniformity throughout the investigation. Participants received consistent instructions, regardless of their group. Minor logistical adjustments to ensure smooth operation during data collection did not affect the trial’s scientific integrity. This manuscript adheres to the CONSORT (Consolidated Standards of Reporting Trials) guidelines for noninferiority trials (Checklist 1) [15].

### Ethical Considerations

The trial was conducted in accordance with the ethical standards of the participating institutions and with the principles of the Declaration of Helsinki. Approval was obtained from the Institutional Review Board at UNICAMP, Brazil (CAAE 79474024.7.0000.5404; approval: 6.893.573; June 18, 2024). Institutional approval was also secured at the collaborating site in the United States prior to participant enrollment. All participants provided informed consent before participation and were assured of confidentiality and the voluntary nature of their involvement. They did not receive any financial compensation or material incentives for their participation in the study.

### Participants

A faculty member not involved in data collection recruited residents of internal medicine and emergency medicine at UNICAMP because they require routine ACLS training. The recruitment of internal medicine and emergency medicine residents from the same institution resulted in a relatively homogeneous participant population, with comparable baseline exposure to ACLS training across groups, consistent with the study’s focus on comparing assessment modalities. Participants in both the traditional in-person group and remote AR group received instruction from UNICAMP faculty instructors, although the AR group’s UNICAMP instructor was remotely located at Stanford’s School of Medicine. Participants were excluded if they had a history of severe motion sickness, current nausea, a history of seizures, or required corrective glasses that would interfere with the use of AR HMD. Participants requiring corrective glasses that interfered with the safe fitting of the AR headset were excluded from the remote AR group due to device compatibility constraints. This exclusion was applied after randomization when headset fitting was attempted. Potential harms, such as physical discomfort, disorientation, or adverse reactions to the AR headset, were predefined and monitored, with participants instructed to report any symptoms immediately during or after the simulation. Recruitment and invitations to participate were conducted through direct oral communication. Data collection occurred in the UNICAMP simulation laboratory.

### Randomization and Blinding

Participants were randomly assigned to either the traditional in-person group or the remote AR group, using a computer-generated randomization sequence (GraphPad Software, Inc). Randomization was performed in a 1:1 allocation ratio to ensure an equal distribution of participants between the 2 groups, without matching or stratification by specialty, year of training, or prior clinical experience. To maintain allocation concealment and minimize selection bias, the randomization sequence was generated by an independent researcher who was not involved in the recruitment, enrollment, or data analysis processes. The independent researcher had no access to participant identities, preserving impartiality in group assignment. Raters of participants’ performance were not blinded to group allocation because ratings were performed live in the simulation setting and the assigned modality was visually apparent. Raters were aware of the study procedures and instruments but were not informed of the expected direction of results. The researchers conducting the statistical analyses were blinded to group assignments, using coded datasets to prevent bias while interpreting results.

### Interventions

After screening, trained research assistants provided informed consent to participants, who then completed demographic questionnaires. Participants were randomly assigned to either the traditional, in-person simulation or the remote-instructor AR simulation. Hereafter, these will be referred to as the traditional in-person group and the remote AR group, respectively. Participants were informed of their group assignments after signing the informed consent form.

To ensure a comparable baseline skill set while preserving the assessment-focused nature of the study, all participants viewed the same brief, scripted 10-minute instructional video immediately before the simulation. This video reviewed core principles of effective team leadership, closed-loop communication, and the VF algorithm, based on the 2020 American Heart Association Guidelines [16]. It did not include hands-on practice, rehearsal, or individualized feedback. This minimal standardization was intended to reduce variability related to prior exposure while avoiding training for the test. Then, each simulation featured 1 participant serving as the team leader, coordinating 3 actors assigned to specific resuscitation roles: chest compressions, ventilation, and defibrillation or medications. These actors were nurses and physicians who acted in scripted supporting roles consistent with standard resuscitation team structures. This design simulated a realistic interprofessional team, consistent with contemporary best practices in resuscitation training [17]. Although the evaluation instrument includes items referring to actions performed by resuscitation team members, these elements were used to operationalize assessment of team leader performance rather than to evaluate individual technical skills. All hands-on tasks were executed by trained actor-participants following predefined scripts, while the team leader was responsible for recognizing deficiencies, issuing corrective instructions, coordinating task allocation, and ensuring adherence to the resuscitation algorithm. The same actors participated in both the AR and traditional scenarios.

The simulation followed a standard, scripted sequence of events simulating an unexpected cardiac arrest (Multimedia Appendix 1) [18]. In both groups, the participant’s role was to lead the resuscitation, including defibrillation and administration of medications such as epinephrine and amiodarone. The instructor facilitated the simulation by controlling the progression of the scenario and updating clinical parameters in real time, in response to the participant’s decisions. The instructor did not provide coaching, feedback, or suggestions regarding clinical actions during the scenario.

In the traditional group, the simulation was facilitated by an instructor who was physically present with the participant in the simulation room. The equipment used included an adult manikin (MegaCode; Laerdal Medical, Inc), a manual defibrillator, a medication cart, a bag-valve-mask, advanced airway equipment, and a stopwatch.

**Figure 1.**
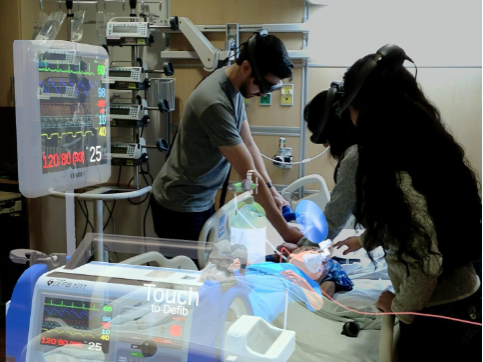
Example view of the augmented reality (AR) simulation environment as seen through the Magic Leap One headset, showing the holographic patient and monitor used during training.

In the remote AR group, research assistants fitted participants with a Magic Leap One (ML1) AR HMD (Magic Leap, Inc). The ML1 used the Chariot AR Medical simulation software (Stanford Chariot Program) which integrates real-time communication into a portable AR medical simulator featuring a holographic patient, monitor, and other medical equipment. The holographic patient’s chest was aligned directly over a real compression torso to provide tactile feedback for the actors. The software has effectively delivered medical simulations in different contexts [12-14,19,20]. Research assistants oriented participants to the ML1 and Chariot AR medical simulation software functionalities before the simulation. Orientation to the AR system required approximately 5 to 10 minutes and included headset fitting, calibration, and a brief walkthrough of the Chariot AR interface and communication features. Each remote AR session used 4 HMDs: 1 ML1 worn by the remote instructor at Stanford and 3 ML1 devices at UNICAMP worn by the team leader and two of the actor-participants. Room requirements were consistent with standard resuscitation simulation: a simulation room with adequate space for the team and equipment, a bed or table to simulate a stretcher, and reliable broadband internet connectivity to support real-time remote facilitation. The participants experienced the same clinical scenario as the in-person group. However, instead of a mannequin, they interacted with a holographic patient and a holographic monitor (Figure 1). In addition, the instructor performed this facilitation role remotely from Stanford, using a headset to interact in real time with participants located in Brazil.

In both groups, the instructor adjusted vital signs, pulses, and other clinical cues in real time according to the participant’s actions. In both cases, the simulation concluded with the return of spontaneous circulation, evidenced by the presence of a pulse and breathing. The scenario had an average duration of 12 (SD 1.1) minutes. At the end, all participants received a structured debriefing and completed the postsimulation questionnaires.

The primary risk associated with the ML1 headset was minor physical discomfort, such as pressure or irritation from wearing the device. To minimize this risk, trained staff ensured proper fitting and adjustment for each participant. The ML1 is a commercially available headset that has undergone manufacturer testing and regulatory review to ensure safety and compliance. Overall, the study posed minimal risk to participants. All participants were informed of their right to withdraw at any time without penalty, preserving their autonomy and comfort throughout the trial.

### Outcomes

Demographic data, collected via Google Forms prior to study initiation, included age, gender, race, level of training, prior exposure to in-person simulations, and prior exposure to AR. The primary outcomes were assessed using two validated instruments: (1) an adapted checklist-based tool, derived from the Evaluation of Advanced Life Support Performance instrument, measuring cognitive leadership and adherence to resuscitation algorithms; and (2) the Behaviorally Anchored Rating Scale (BARS), evaluating observable nontechnical behaviors.

For the adapted checklist, we calculated 2 types of scores for each participant—objective and subjective—based on the original instrument’s 26-item checklist and global rating scale (Multimedia Appendix 1) [4]. Although originally developed to assess both technical and nontechnical skills, in this study, the instrument was adapted specifically to evaluate cognitive performance and scenario leadership, such as clinical decision-making, adherence to resuscitation protocols, and effective team coordination. No psychomotor or procedural technical skills were assessed, as all hands-on resuscitation tasks were performed by trained health care professional actors under the direction of the participant. Accordingly, we excluded checklist elements that require direct evaluation of team members’ psychomotor execution (eg, chest compression and ventilation technique or quality metrics). We retained items that can be attributed to the team leader’s cognition and directives, including recognition and prioritization of actions, algorithm adherence (eg, rhythm recognition or defibrillation and medication timing), situational awareness, and team coordination or closed-loop communication. The checklist items, each scored from –2 to +2, covered two domains: (1) adherence to cardiopulmonary resuscitation guidelines and (2) clinical decision making. The objective score was obtained by summing the ratings across the 26 items, reflecting the participant’s ability to manage the crisis effectively. The subjective score was a global rating from 0 to 10, assigned by the rater at the end of the simulation to summarize overall performance. These 2 scores were independently assigned by 2 trained raters during each session, and final scores were calculated as the average of both raters’ assessments.

BARS is a tool designed to assess observable behavioral competencies and combines qualitative and quantitative assessment by linking specific behavioral examples to numerical ratings. The four domains of this instrument are: (1) vigilance situation-aware, (2) decision-making, (3) communication, and (4) teamwork (Multimedia Appendix 1) [21]. BARS rates these domains as “poor,” “average,” or “excellent” and assigns a score ranging from 1 to 9. Two trained raters assessed behavioral competencies using the BARS scale in real time during and immediately after each simulation session, offering a complementary perspective on participants’ nontechnical performance. The BARS domain scores were prespecified as secondary noninferiority outcomes to provide supportive evidence on nontechnical leadership performance.

Assessments were completed live during each simulation session by 2 trained raters, and no video was recorded. Prior to data collection, raters completed 3 calibration sessions (trial runs) in which they practiced scoring using the adapted checklist and BARS, reviewed the intent of each item and domain, and discussed discrepancies with the research team to standardize scoring.

The secondary outcomes included usability and ergonomic perceptions of the AR system for participants in the AR group. Usability was assessed using the system usability scale (SUS; Multimedia Appendix 1) [22]. The SUS includes 10 items; each rated on a 5-point Likert scale from 1 (“Completely Disagree”) to 5 (“Completely Agree”). Ergonomics were measured using the International Organization for Standardization (ISO) 9241‐400 scale (Multimedia Appendix 1) which is composed of 6 items, each rated on a 5-point Likert scale from 1 (“Totally Disagree”) to 5 (“Totally Agree”). AR group participants completed these questionnaires at the end of the simulation.

### Sample Size

A typical mean score in the Evaluation of Advanced Life Support Performance instrument during reasonable simulation performance is 7.875, with an IQR from 7 to 8.75 [18]. Importantly, this estimate was derived from the instrument’s 0 to 10 subjective or global rating scale, which was retained unchanged in our adapted version; thus, the sample size calculation does not depend on the adapted objective checklist score (0‐52). Given a noninferiority margin of 20% and assuming a SE of 1.3, 16 participants (8 in each group) were needed to achieve 80% power with a 1-sided 95% CI. To account for incomplete data and dropouts, a total of 30 participants (15 in each group) was set as the minimal enrollment target. However, given that participants were recruited from a single institution’s residency program, no upper limit was imposed on enrollment. All interested volunteers were allowed to participate, even after the minimum sample size required for statistical power had been reached.

### Statistical Analysis

The primary analysis was per-protocol and included all randomized participants who completed the assigned simulation session with a fully evaluable checklist and BARS outcomes. Participants who did not attend the scheduled session had no outcome measurements and were not included in outcome analyses.

We conducted a noninferiority analysis using a 20% noninferiority margin based on the in-person group’s mean score. Because there is no established minimally important educational difference for the adapted checklist or BARS in ACLS leadership assessment, we prespecified a 20% noninferiority margin as a pragmatic threshold, based on consensus among the study team’s ACLS instructors, to define acceptable similarity to the in-person benchmark. Noninferiority was established if the lower bound of the 95% CI for the AR group’s mean score exceeded this threshold. Performance scores from 2 independent raters were averaged for analysis, and interrater reliability (IRR) was assessed using the intraclass correlation coefficient (ICC). ICC values between 0.50 and 0.75 were considered moderate, 0.75 to 0.90 as good, and values above 0.90 as excellent [23]. We did not compute internal consistency indices (eg, Cronbach α) because the checklist is a structured performance rubric spanning heterogeneous actions. We therefore report IRR and coherence between objective and global scores. BARS domain scores were computed at the participant level by averaging the 2 raters’ ratings. Noninferiority for each domain was evaluated using the prespecified 20% margin relative to the traditional group mean, based on 95% CIs. We did not perform superiority hypothesis testing (no *P* values or standardized effect sizes). Usability and ergonomics ratings were analyzed using descriptive statistics for the AR group only. All analyses were performed in RStudio (RStudio Team). No ancillary analyses were conducted.

## Results

### Participant Flow and Baseline Characteristics

**Figure 2.**
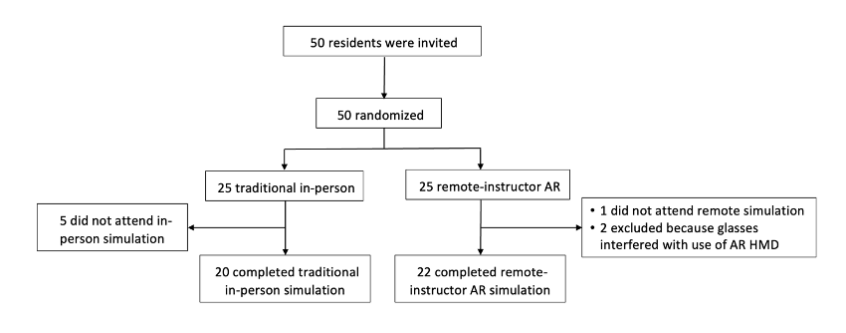
CONSORT (Consolidated Standards of Reporting Trials) flow diagram. AR: augmented reality; HMD: head-mounted display.

We invited 50 residents to participate. Of these, 42 met the inclusion criteria, agreed to participate, and completed all stages and questionnaires of the study. We randomized participants into 2 groups: 20 (48%) in the traditional group and 22 (52%) in the remote AR group. The flow diagram of the participants is in Figure 2. Participants in both groups were at comparable stages of postgraduate training, as reflected by similar distributions of specialty and recent ACLS exposure (Table 1).

**Table 1.** Baseline demographic characteristics of participants in the traditional in-person group and remote augmented reality (AR) group.

Characteristics	Traditional (n=20)	Remote AR (n=22)
Demographics^a^		
Age, mean (SD)	26.8 (1.9)	27.1 (2.3)
Female, n (%)	11 (55)	11 (50)
Specialty, n (%)		
Internal medicine	18 (90)	18 (81.8)
Emergency medicine	2 (10)	4 (18.2)
Time since last ACLS^b^ course, n (%)		
Never taken	0 (0)	1 (4.5)
<1 year	5 (25)	6 (27.3)
<2 years	8 (40)	7 (31.8)
<3 years	4 (20)	4 (18.2)
>3 years	3 (15)	4 (18.2)
How much do you work in emergency settings (1=“little/not at all” to 5=“a lot/routinely”), mean (SD)	3.8 (1.5)	3.9 (1.2)
Have you ever used augmented reality in medical education? (yes=1 and no=0), n (%)	0 (0)	0 (0)

a For continuous variables, values are presented as mean (SD); for categorical variables, values are presented as n (%).

b ACLS: advanced cardiac life support.

### Primary Outcome: Team Leader Performance

The remote AR group demonstrated comparable performance to the traditional group in cognitive leadership tasks, in the objective and subjective scores (Table 2). In all cases, the lower bound of the 95% CI for the AR group exceeded the predefined noninferiority margin, confirming that this group performed noninferiorly to the traditional in-person simulation. IRR was good, with an ICC of 0.84 for the objective score and 0.76 for the subjective score. Additionally, the correlation between the 2 types of scores was 0.855 (95% CI 0.745‐0.920), indicating good internal coherence between checklist-based performance and global assessments.

**Table 2.** Scores from the adapted checklist from Evaluation of Advanced Life Support Performance, compared across simulation modalities with noninferiority thresholds.

Outcome	Traditional, mean (SD)	Remote AR^a^, mean (SD)	Noninferiority margin^b^	95% CI (AR)
Objective score^c^	42.6 (5.8)	41.6 (6.2)	34.1	38.9-44.4
Subjective score^d^	7.9 (1.0)	8.1 (1.2)	6.3	7.6-8.6

a AR: augmented reality.

b Noninferiority cutoffs were defined as 20% below the in-person group mean for each outcome.

c Objective checklist score range = −52 to +52 (26 items scored −2 to +2). Higher scores indicate better performance. Subjective/global score range = 0-10. Higher scores indicate better overall performance. Noninferiority cutoffs were defined as 20% below the in-person group mean for each outcome.

d Subjective/global score range = 0-10. Higher scores indicate better overall performance.

The remote AR group demonstrated performance comparable to the in-person group using BARS (Table 3). For each BARS domain, the lower bound of the 95% CI for the AR group exceeded the predefined noninferiority margin, indicating noninferior performance relative to traditional in-person simulation.

**Table 3.** Leader behavior performance assessed with the Behaviorally Anchored Rating Scale (BARS), compared across simulation modalities with noninferiority thresholds^a^.

Outcome	Traditional, mean (SD)	Remote AR^b^, mean (SD)	Noninferiority margin	95% CI (AR)
Vigilance (BARS)	8.2 (0.7)	8.3 (0.7)	6.5	8.0-8.6
Decision-making (BARS)	8.2 (0.8)	8.3 (0.9)	6.5	7.9-8.7
Communication (BARS)	8.2 (0.8)	8.1 (0.9)	6.6	7.8-8.5
Teamwork (BARS)	8.3 (0.7)	8.4 (0.6)	6.6	8.1-8.7

a BARS domain scores range from 1 to 9; higher scores indicate better performance. Noninferiority cutoffs were defined as 20% below the in-person group mean for each outcome.

b AR: augmented reality.

### Secondary Outcome: Usability

**Figure 3.**
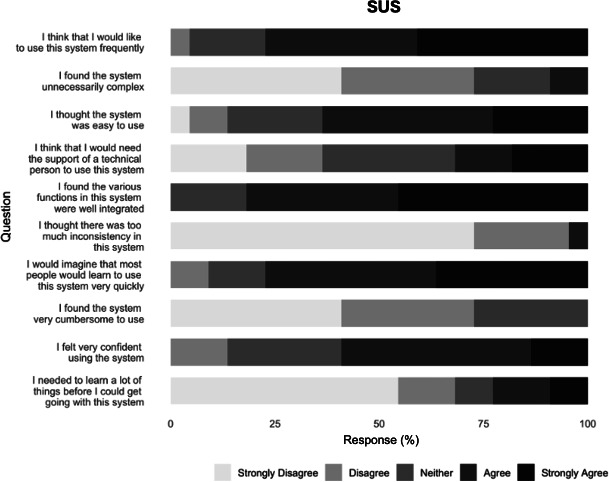
System usability scale (SUS) responses for the augmented reality (AR) simulation system (n=22).

We computed the average overall SUS score (0‐100), which was 73.8 (SD 13.4) among AR participants (n=22), consistent with good or acceptable usability. Most participants agreed or strongly agreed that the AR system was easy to use, well-integrated, and worth frequent use, while they disagreed with statements about complexity, inconsistency, and cumbersomeness (Figure 3). Only the item regarding the need for technical support drew mixed opinions. Overall, the distribution indicates high perceived usability of the platform.

### Secondary Outcome: Ergonomics

**Figure 4.**
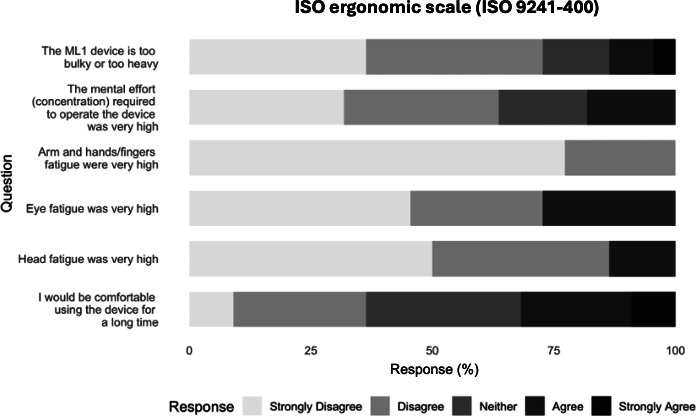
International Organization for Standardization (ISO) 9241‐400 ergonomic responses for the augmented reality (AR) headset (n=22). ML1: Magic Leap One.

Regarding ergonomics, a majority rejected statements about excessive bulk, fatigue, or high mental effort, and most agreed the headset would be comfortable for extended use (Figure 4). The responses support minimal physical or cognitive burden during simulation.

No AR-related adverse effects (eg, nausea, dizziness, or eye strain) were observed or reported during or immediately after the simulation sessions, and no participant discontinued the session due to headset discomfort. Minor connectivity lag was noted at the beginning of some remote AR sessions, but no session required termination or rescheduling, and all simulations were completed as planned.

## Discussion

### Principal Findings

These findings suggest that remote AR simulation may serve as a feasible alternative to traditional in-person simulation for assessing team leader performance during cardiac arrest scenarios. Beyond demonstrating noninferior outcomes, this study highlights the practicality of AR for remote assessment, characterized by high usability, minimal technical barriers, and reliable real-time instruction. Our findings align with evidence from systematic reviews and meta-analyses demonstrating the feasibility of AR and VR simulations in medical education [9,24,25]. While much of this literature focuses on training applications, our study extends these findings by showing that AR can also serve as a standardized assessment modality, producing performance outcomes comparable to conventional in-person simulation.

Evidence suggests that AR can preserve key elements of team communication, including unbroken eye contact, while enabling performance outcomes comparable to those seen in conventional manikin-based simulations [12,26,27]. In addition, AR can deliver real-time clinical prompts and integrate simulated patient data to support decision-making during assessment scenarios [28]. While current evidence does not consistently demonstrate superiority over traditional methods of basic and advanced life support training, our findings contribute to the growing recognition of immersive technologies as promising and scalable alternatives for resuscitation education and assessment across diverse contexts [24].

Video conferencing has also enabled remotely instructed simulations. For example, remote neonatal training showed noninferior technical skills at 2 months among inexperienced medical students, supporting its use in resource-limited settings [29]. A key strength of our study was its cross-continental, parallel group, noninferior randomized design testing instructor-led AR simulation for team leader performance in a simulated cardiac arrest scenario. By delivering cross-continental remote facilitation from Stanford while conducting all learner assessments at UNICAMP, this study serves as a proof of concept for international remote AR assessment. Broader generalizability across settings and learner populations will require replication in additional sites. The required setup (4 headsets, a compression torso, and broadband internet) directly addressed logistical barriers noted in implementation reviews [5]. This portability makes AR simulation especially relevant for rural hospitals, humanitarian settings, and low- and middle-income countries with limited access to simulation centers [9,24].

While this study supports the feasibility of using remote AR simulation for assessing team leader performance, it is important to critically consider whether this level of technological immersion is necessary for the intended evaluation goals. Desktop simulations, video-enhanced medical scenarios, and computer-based training modules are well-established, low-cost alternatives that can effectively assess decision-making and communication in certain contexts, particularly for single-provider scenarios [30-33]. These approaches are scalable, accessible in low-resource environments, and do not require specialized hardware or technical support. In settings where only cognitive rehearsal or basic assessment is required, they may represent more pragmatic solutions. Our study introduces an additional modality of simulation-based assessment, focusing specifically on team leader performance. Such an approach could be particularly relevant for certification processes in which demonstration of leadership competence is a required component.

The rationale for using AR in this study was to replicate not only the cognitive demands but also the spatial awareness and real-time team coordination involved in resuscitation leadership. AR can integrate contextual cues, spatial orientation, and immersive team interactions that are more representative of the clinical environment than screen-based tools. Unlike video-enhanced medical scenarios, which are typically linear and observer-based, AR allows for active, embodied participation in real-time decision-making within a simulated physical space, thereby enhancing the ecological validity of assessment. This immersive approach supports evaluation of leadership behaviors such as spatial coordination, situational awareness, and communication under time pressure. Moreover, because only the team leader, defibrillation operator, and remote instructor require HMDs, the same hardware can serve larger cohorts through staggered sessions or observer roles, lowering per learner costs compared to setups using multiple high-fidelity mannequins. After initial capital costs, software and headset upkeep are more affordable than ongoing maintenance of complex mannequins [34]. As broadband access expands, AR-enabled simulation provides a scalable option for delivering standardized clinical assessments in underserved settings [9].

Participants generally rated ergonomics and usability as adequate. This was encouraging, as prior studies have reported nausea, dizziness, and visual discomfort after short VR or AR sessions [26,27,35]. Unlike VR, AR reduces motion sickness by allowing natural visual grounding [35]. In our study, scenarios lasted around 12 minutes, likely minimizing adverse effects. Continued improvements in headset design may also have contributed.

Although AR is less established than in-person or VR simulation, it offers potential educational benefits by integrating digital content with the physical environment, enabling learners to interact with both holographic and real clinical tools. This combination may enhance immersion and support knowledge transfer, particularly in scenarios requiring spatial awareness and team coordination. As AR technologies become more accessible, they could serve as a complementary option within a spectrum of simulation-based assessment modalities, particularly where portability and cost efficiency are priorities. Continued research is needed to define its role, establish validity evidence, and determine how AR-based assessments relate to long-term clinical performance and patient outcomes.

### Limitations

This study had several limitations. First, recruitment at a single institution limits external validity, and replication at other sites is needed. Second, enrolling only residents restricts generalizability to other learner groups, especially medical students. Third, usability and ergonomic data were self-reported, which may introduce response bias despite the use of validated scales. Fourth, the study evaluated performance at a single time point and therefore does not provide information on longitudinal consistency or reproducibility of assessment outcomes. Fifth, 2 participants were excluded after randomization due to incompatibility between corrective glasses and the AR headset. This exclusion applied only to the AR group and reflects a technical device limitation rather than participant characteristics. Sixth, cardiopulmonary resuscitation technical quality metrics (eg, compression depth, rate, and recoil) were not measured; therefore, our findings speak to team leader cognition and nontechnical behaviors (decision-making, communication, and coordination) rather than hands-on resuscitation technical performance. Seventh, the study used a single VF scenario, which may not capture the full range of skills needed for real-world cardiac arrest. Finally, because assessments were conducted live and the simulation modality was visually apparent, raters were not blinded to group allocation, which may have introduced observer bias in favor of or against the AR modality.

While this study focused on team leader performance, future research should incorporate additional outcomes such as chest compression quality and other key resuscitation tasks. Expanding to diverse scenarios would strengthen the ecological validity of AR-based assessment. While acknowledging that role rotation, common in ACLS training, was not implemented, further studies could examine whether AR platforms are suitable for assessing competence across multiple roles. Future studies should explore how AR platforms can provide real-time, objective metrics to support standardized, instructor-independent evaluations. These innovations may enable scalable, competency-based credentialing in settings with limited faculty resources. Further research is also needed to determine whether these immersive features confer measurable advantages over simpler screen-based approaches when the primary goal is leadership-focused assessment.

### Conclusions

This international noninferiority randomized trial found that remote AR simulation produced team leader performance comparable to traditional in-person simulation in a VF arrest scenario. Usability scores were high and ergonomic burden was low, indicating that learners operated the headset comfortably and with minimal technical support. These findings suggest that remote instructor-led AR simulation may be a feasible option for delivering standardized, team-based assessment of cardiac arrest leadership in contexts where geographic, logistical, or financial constraints limit access to conventional simulation.

## Supplementary material

10.2196/84367 Multimedia Appendix 1 Standardized simulation script for cardiac arrest scenario.

10.2196/84367 Checklist 1 CONSORT checklist.

## Abbreviations

ACLS: advanced cardiac life support
AR: augmented reality
BARS: Behavioral Assessment Rating Scale
CONSORT: Consolidated Standards of Reporting Trials
HMD: head-mounted display
ICC: intraclass correlation coefficient
IRR: interrater reliability
ISO: International Organization for Standardization
SUS: system usability scale
VF: ventricular fibrillation
VR: virtual reality
