# Supplementary material for: Remote Augmented Reality Versus Traditional Simulation for Team Leader Assessment in a Cardiac Arrest Scenario: Noninferiority Randomized Controlled Trial
Source: JMIR Med Educ. 2026 Mar 16;12:e84367. doi: 10.2196/84367 (PMC12991189; doi:10.2196/84367)
Supplement: Multimedia Appendix 1 [file mededu-v12-e84367-s001.docx]

**Supplemental Materials**

**Supplemental Material A - Standardized Simulation Script for Cardiac Arrest Scenario**

**[SIMULATION SCENARIO DESIGN]**

**BRIEFING:**

You are the leader of a Rapid Response Team (RRT) at a university hospital. This team includes 2 additional physicians and a nurse who already know their roles during the response. The scenario takes place in an emergency department. Say findings and your actions during the response. You will all already be wearing procedure gloves.

**Case:** A 65-year-old man with a history of type 2 diabetes mellitus is admitted to the ED with malaise lasting one hour and chest discomfort lasting 40 minutes. The patient has no fever and his mental status is normal.

The 12-lead ECG shows sinus tachycardia with prominent ST segment depression in the anterior precordial leads. He has not received any medication so far, but already has intravenous access. The RRT is immediately requested to participate in the care, as his hemodynamic status is unstable. Shortly after the initial assessment, the patient suddenly deteriorates, becoming unresponsive and pulseless, which prompts immediate initiation of basic life support according to standard resuscitation protocols.

**RESCUER ROLES:**

**RESCUER 1: Chest compressions / Airway (will not use headset)**

- Starts compressions incorrectly: SLOW (<100 comp/min) and WEAK (<5 cm depth): the intention is for the leader to identify and provide feedback to correct compression quality while the defibrillator arrives.
- Alternates with Rescuer 2 every cycle.

**RESCUER 2: Airway / Chest compressions (will not use headset)**

- Performs ventilations (30:2) correctly (1s for each ventilation).
- Alternates with Rescuer 1 every cycle.

**RESCUER 3: Medication and Defibrillation (will use headset)**

- Reports going to get the defibrillator (wait 1min and 20s to arrive with defibrillator). The instructor will indicate the correct moment.

**SCENARIO PROGRESSION**

| Times | Actions |
| --- | --- |
| 0:00 | RRT arrival |
| 0:10 | Responsiveness/breathing check |
| 0:20 | Help/defibrillation requested |
| 0:30 | Pulse check |
| 0:40 | CPR initiation |
| 2:00 | Defibrillator arrival |
| 2:10 | Rhythm analysis |
| 2:20 | **Shock delivery** |
| 2:30 | CPR initiation |
| 4:30 | Rhythm analysis |
| 4:40 | **Shock delivery** |
| 4:50 | CPR initiation + epinephrine |
| 6:50 | Rhythm analysis |
| 7:00 | **Shock delivery** |
| 7:10 | CPR initiation + amiodarone/lidocaine |
| 9:10 | Rhythm analysis |
| 9:20 | Organized rhythm recognition |
| 9:30 | Pulse check |
| 9:40 | Post-cardiac arrest care |
| 10:00 | End of case |

The scenario was structured to generate predefined leadership decision points rather than relying on spontaneous team errors. At the start of the simulation, Rescuer 1 intentionally performed chest compressions with inadequate quality (slow rate and insufficient depth), requiring the team leader to recognize the deficiency and provide corrective feedback. This element was directly linked to checklist items assessing leadership oversight and adherence to resuscitation guidelines.

Throughout the scenario, all key actions, including requesting compressor rotation, determining whether defibrillation was indicated, selecting the appropriate energy dose, instructing the team to clear before shock delivery, and directing the immediate resumption of chest compressions, were initiated by the team leader. These actions required continuous decision-making, situational awareness, and effective verbal communication. The scripted roles of the rescuers were consistent across both simulation modalities and were designed to ensure that leadership and communication competencies could be reliably observed and assessed.

**Supplemental Material B - Portuguese version of the Instrument for the Evaluation of Advanced Life Support Performance. This tool assesses technical and clinical decision-making skills during ACLS simulations, using a 26-item scale scored from -2 to +2 across key performance domains.**

**EVALUATION FORM DATE: ____/____/ 2024**

☐ TRADITIONAL Simulation

☐ AUGMENTED REALITY Simulation

**Participant name (scenario leader):**

**Rescuer 1:_________________________** **Rescuer 2:________________________**

**Rescuer 3:__________________________**

**Evaluator:_____________________________________________________________**

| **QUESTION** | **EVALUATION CRITERIA** |
| --- | --- |

**1. GUIDELINE ADHERENCE**

**A. Recognition of the need for cardiopulmonary resuscitation**

**1. Are patient responsiveness and breathing checked?** *Patient responsiveness and breathing should be checked at the beginning of care.*

| Score | Criteria |
| --- | --- |
| +2 | Checked responsiveness **AND** breathing. |
| 0 | Did not check responsiveness **OR** did not check breathing. |
| -2 | Did not check responsiveness **AND** did not check breathing. |

**2. Was help requested immediately?** *Help should be requested after recognizing the need for CPR.*

| Score | Criteria |
| --- | --- |
| +2 | Help was requested immediately **AND** included request for a defibrillator. |
| 0 | Help was requested, but did **NOT** include request for a defibrillator. |
| -2 | Help was **NOT** requested and a defibrillator was **NOT** requested. |

**3. Was pulse checked?** *Pulse should be checked in a central region (carotid/femoral) within 10s.*

| Score | Criteria |
| --- | --- |
| +2 | Pulse was checked within 10 s. |
| 0 | Pulse was checked, but was **NOT** a central pulse. |
| -2 | Pulse was not checked within 10 s. |

**4. Were chest compressions started immediately?** *This should be concluded within a maximum of 10 s. after checking pulse. After 1 minute the instructor will say that the patient is monitored.*

| Score | Criteria |
| --- | --- |
| +2 | Chest compressions were started within 10 s. |
| -2 | Chest compressions were not started within 10 s. |

**B. Chest compression quality (after 1:30 min of 1st cycle the instructor informs that the patient is MONITORED)**

**5. Was hand and arm position correct?** *Adults: compressions are performed in the middle of the sternum with the palm of one hand at the base and the other hand over it, with arms extended and shoulders perpendicular above the patient's thorax.*

| Score | Criteria |
| --- | --- |
| +2 | The compressor had correct hand position **AND** correct compression angle. |
| 0 | The compressor had incorrect hand position **OR** incorrect compression angle. |
| -2 | The compressor had incorrect hand position **AND** incorrect compression angle. |

**6. Was the compression rate 100 to 120 per minute?** *The correct compression rate is 100-120 per minute for all patients. Therefore, in 20s one should perform 33 to 40 compressions.*

| Score | Criteria |
| --- | --- |
| +2 | All compressors had the correct compression rate. |
| +1 | Most compressors had the correct compression rate. |
| -1 | Most compressors had an incorrect compression rate. |
| -2 | All compressors had an incorrect compression rate. |

**7. Was the compression-ventilation rate correct?** *The correct compression-ventilation rate for adult patients is 30:2.*

| Score | Criteria |
| --- | --- |
| +2 | The compression-ventilation rate was correct all the time. |
| +1 | The compression-ventilation rate was correct most of the time. |
| -1 | The compression-ventilation rate was incorrect most of the time. |
| -2 | The compression-ventilation rate was incorrect all the time. |

**8. Was compression depth correct?** *The correct compression depth in adults is 5 to 6 cm of thorax. This item should be checked in the first cycle.*

| Score | Criteria |
| --- | --- |
| +2 | All had correct compression depth. |
| +1 | Most had correct compression depth. |
| -1 | Most had incorrect compression depth. |
| -2 | All had incorrect compression depth. |

**9. Was the number of interruptions in chest compressions minimized?** *The number of interruptions in chest compression should be minimized.*

| Score | Criteria |
| --- | --- |
| +2 | All interruptions were necessary. |
| +1 | Few unnecessary interruptions occurred. |
| -1 | Many unnecessary interruptions occurred. |
| -2 | Unnecessary interruptions occurred in all compression-ventilation cycles. |

**10. Was compressor rotation adequate?** *Compression rotation is recommended to avoid poor quality compressions due to rescuer fatigue, for example, every 2-4 min.*

| Score | Criteria |
| --- | --- |
| +2 | The compressor rotated every 2-4 min or when necessary. |
| +1 | The compressor generally rotated every 2-4 min. |
| -1 | The compressor rarely rotated every 2-4 min. |
| -2 | The compressor did not rotate even when necessary. |

**11. Did the team leader give continuous corrective feedback about CPR quality?** *The team leader should continuously give corrective feedback about CPR quality to the team, to improve CPR quality.*

| Score | Criteria |
| --- | --- |
| +2 | The team leader gave corrective feedback about CPR quality to the team **OR** no evident inadequacy was observed. |
| +1 | The team leader gave corrective feedback about CPR quality **most** of the time. |
| -1 | The team leader did **NOT** give corrective feedback about CPR quality **most** of the time. |
| -2 | The leader gave no corrective feedback about CPR quality. |

**C. Ventilation quality**

**12. Was oxygen administered as quickly as possible?** *100% oxygen should be administered throughout the cardiac resuscitation process.*

| Score | Criteria |
| --- | --- |
| +2 | 100% oxygen was administered from beginning to end. |
| +1 | 100% oxygen was administered during most of the process, but not from beginning to end. |
| -1 | 100% oxygen was administered in less than half of the resuscitation process. |
| -2 | 100% oxygen was not administered. |

**13. Was the airway secured with appropriate equipment until the end of the scenario?** *The airway should be secured throughout the resuscitation process. The choice of equipment (mask, oropharyngeal airway, supraglottic advanced airway, endotracheal intubation) depends on the skill of the professionals on site. Intubation can be performed during continuous chest compressions.*

| Score | Criteria |
| --- | --- |
| +2 | The airway was secured throughout the resuscitation process and an advanced airway was inserted by the end of the scenario. |
| 0 | The airway was secured most of the time and an advanced airway was not inserted by the end of the scenario. |
| -2 | The airway was not secured and an advanced airway was not inserted by the end of the scenario. |

**D. Rhythm control and defibrillation quality**

**14. Was the rhythm (VF, pVT) correctly identified?** *The rhythm (VF, pVT) should be correctly identified.*

| Score | Criteria |
| --- | --- |
| +2 | The rhythm was correctly identified in all situations. |
| +1 | The rhythm was correctly identified in most situations. |
| -1 | The rhythm was incorrectly identified in most situations. |
| -2 | The rhythm was incorrectly identified in all situations. |

**15. Was the shock applied immediately and with the correct energy?** *The shock should be applied immediately after VF/pVT identification with the correct energy (adults 200 J biphasic) / maximum energy.*

| Score | Criteria |
| --- | --- |
| +2 | The shock was applied immediately after identifying the shockable rhythm **AND** with the correct energy. |
| +1 | The shock was not applied immediately, but the energy was correct. |
| -1 | The shock was applied immediately, but the energy was wrong. |
| -2 | The shock was not applied immediately and the energy was wrong when applied. |

**16. Was everyone instructed to stand clear BEFORE applying the shock?** *Before applying the shock, the leader should verbalize for everyone to stand clear of the patient.*

| Score | Criteria |
| --- | --- |
| +2 | It was verbalized to stand clear of the patient before ALL shocks applied. |
| +1 | It was verbalized to stand clear of the patient before **most** shocks applied. |
| -1 | It was verbalized to stand clear of the patient before **few** shocks applied. |
| -2 | It was NOT verbalized to stand clear of the patient before all shocks applied. |

**17. Were chest compressions resumed immediately after the shock?** *Chest compressions should be resumed within a maximum of 5 seconds after shock delivery.*

| Score | Criteria |
| --- | --- |
| +2 | Chest compressions were resumed immediately after the shock. |
| -2 | Chest compressions were NOT resumed immediately after the shock. |

**18. Was rhythm analysis performed at 2-minute intervals?** *Rhythm analysis should be performed at 2 min intervals (+/- 10 s.).*

| Score | Criteria |
| --- | --- |
| +2 | Rhythm analysis was performed at 2 min intervals (+/- 10 s.) all the time. |
| +1 | Rhythm analysis was performed at 2 min intervals (+/- 10 s.) most of the time. |
| -1 | Rhythm analysis was not performed at 2 min intervals (+/- 10 s.) most of the time. |
| -2 | Rhythm analysis was not performed at 2 min intervals (+/- 10 s.) at any time. |

**E. Medication and fluid therapy**

**19. Was epinephrine use adequate?** *Resuscitation medications include epinephrine and amiodarone. Medication use refers to timing, dose, and route of administration of a given medication. Adults receive a dose of 1 mg of epinephrine every 3--5 minutes if the rhythm is VF/pVT. After the second refractory shock, initiation of epinephrine administration is recommended.*

| Score | Criteria |
| --- | --- |
| +2 | Epinephrine use was at adequate time AND correct dose. |
| 0 | Epinephrine use was at adequate time OR correct dose (but not both). |
| -2 | Epinephrine use was at **wrong** time and **incorrect** dose. |

**20. Was amiodarone use adequate?** *After the third cycle of shock and compression, adults receive a dose of 300 mg of amiodarone and 150 mg every 3--5 minutes if VF/VT rhythm continues. Lidocaine (150 mg) can be used if amiodarone is not available.*

| Score | Criteria |
| --- | --- |
| +2 | Amiodarone use was at adequate time AND correct dose. |
| 0 | Amiodarone use was at adequate time OR correct dose (but not both). |
| -2 | Amiodarone use was at **wrong** time and **incorrect** dose. |

**F. Return of spontaneous circulation**

**21. Did the leader check pulse after the patient presented organized rhythm?** *During rhythm analysis, if the patient presents an organized rhythm on the monitor it is necessary to check the central pulse to confirm return of spontaneous circulation and exclude diagnosis of PEA.*

| Score | Criteria |
| --- | --- |
| +2 | Pulse was checked for 5 to 10 seconds. |
| 0 | Pulse was checked, but NOT for 5 to 10 seconds. |
| -2 | Pulse was not checked. |

**22. Post-arrest care** *Post-arrest care will consist of these 3 items: a) Check/verbalize* ***vital signs****; b) Request* ***ECG****; and c) Request* ***chest X-ray****.*

| Score | Criteria |
| --- | --- |
| +2 | All 3 items were requested. Specify: |
| +1 | Only 2 items were requested. Specify: |
| -1 | Only 1 item was requested. Specify: |
| -2 | None of the items were requested. |

**2. CLINICAL DECISION MAKING**

**23. Were tasks prioritized adequately?** *Tasks should be prioritized adequately according to guidelines (for example, prioritizing compressions and defibrillation before insertion of advanced airway and medication administration).*

| Score | Criteria |
| --- | --- |
| +2 | Tasks were prioritized adequately all the time. |
| +1 | Tasks were prioritized adequately most of the time. |
| -1 | Tasks were prioritized inadequately most of the time. |
| -2 | Tasks were prioritized inadequately all the time. |

**24. Did the team leader continuously maintain overall situation awareness?** *The team leader should continuously maintain overall situation awareness.*

| Score | Criteria |
| --- | --- |
| +2 | The team leader maintained overall situation awareness all the time OR no evident inadequacy was noted. |
| +1 | The team leader maintained overall situation awareness most of the time. |
| -1 | The team leader did not maintain overall situation awareness most of the time. |
| -2 | The team leader did not maintain overall situation awareness. |

**25. Did the team leader continuously inform others when it was time for an intervention according to the resuscitation algorithm?** *A team member should continuously inform others when it is time for an intervention according to the resuscitation algorithm (for example, rhythm control).*

| Score | Criteria |
| --- | --- |
| +2 | A team member informed others when it was time for an intervention according to the resuscitation algorithm all the time OR no evident inadequacy was observed. |
| +1 | A team member informed others when it was time for an intervention according to the resuscitation algorithm most of the time. |
| -1 | A team member did not inform others when it was time for an intervention according to the resuscitation algorithm most of the time. |
| -2 | A team member did not inform others when it was time for an intervention according to the resuscitation algorithm. |

**26. Was "closed-loop communication" used?** *"Closed-loop" communication should be used in cardiac resuscitation to ensure understanding of orders and improve communication. Requests should be directed to specific team members. Closed-loop communication means that the receiver of a message repeats the message back to the initiator, who in turn confirms that the message was correctly understood.*

| Score | Criteria |
| --- | --- |
| +2 | Closed-loop communication was used and orders were directed to specific team members all the time. |
| +1 | Closed-loop communication was used and orders were directed to specific team members most of the time. |
| -1 | Closed-loop communication was not used and orders were not directed to specific team members most of the time. |
| -2 | Closed-loop communication was not used and orders were not directed to specific team members. |

**TOTAL POINTS SCORE: _______________**

**Your subjective score (0-10): _______________**

**Supplemental Material C - Behaviorally Anchored Rating Scale (BARS) for Non-Technical Skills. This instrument evaluates vigilance, decision-making, communication, and teamwork using qualitative behavioral descriptors and a 1-9 numerical scale.**

**BEHAVIORAL/NON-TECHNICAL SKILLS RATING SYSTEM (BNTSRS)**

In addition to the technical performance elements, the BNTSRS will assess the behavioral skills, or non-technical skills (NTS), of the participant featured in the scenario. The four NTS performance categories to be evaluated are: vigilance/awareness, dynamic decision-making and task management, communication, and teamwork.

**Evaluators must observe the entire scenario before making NTS and holistic evaluations.** Equal weight should be given to behaviors throughout all periods of the scenario, and evaluators should be careful not to be influenced by initial behaviors ("halo effect") or final behaviors (most recent in memory).

The BNTSRS uses a Behaviorally Anchored Rating System (BARS) to score NTS. BARS is based on the MATRIX shown on the following page that details the aspects to be considered when making each evaluation. Below is an excerpt from the "AHRQ MOCA Assessment Form" itself showing how the evaluator would then record the evaluations, using terms and numbers that correspond to those shown in the MATRIX.

**
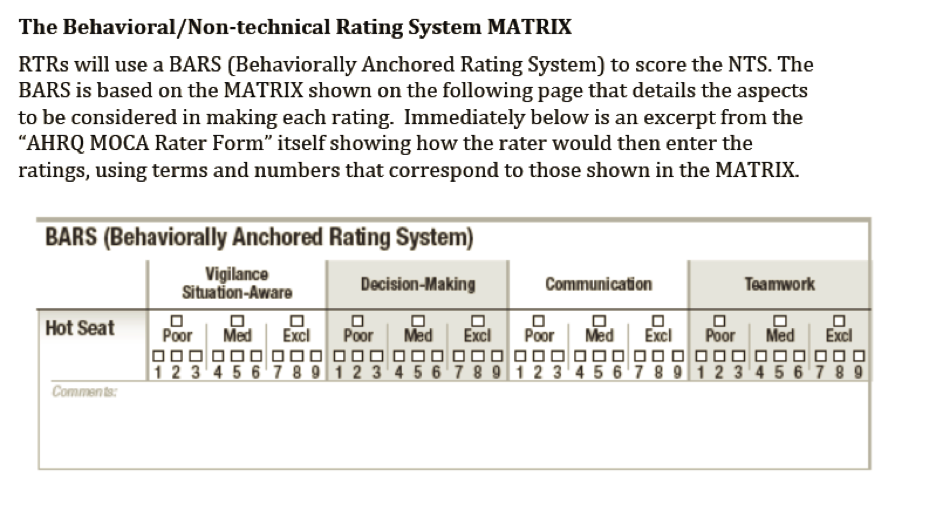
**

**(PLEASE LOOK AT THE BARS MATRIX ON THE FOLLOWING PAGE)**

The first row shows the **4 CATEGORIES** of Behavioral/Non-Technical performance:

- Vigilance/Situation Awareness
- Dynamic Decision-Making and Task Management (Abbreviated as 'Decision-Making')
- Communication
- Teamwork

The second row of the NTS Assessment Matrix (BARS) provides a set of DESCRIPTORS for three general performance levels for each of the CATEGORIES ("Poor, Average, and Excellent"). We call a general performance level a 'BIN.'

The third row shows that within each BIN there are three possible numbers that can be chosen as a sub-score for evaluating the participant's performance in that BIN. These sub-scores can be considered as adding a "-", neutral, or "+" to the grade corresponding to that BIN.

The matrix cells describe the types of performance elements for a given CATEGORY that would place someone's performance evaluation in that BIN. The lists of performance items in each descriptor are presented as examples. They should NOT be evaluated individually, nor will all be present or observable for any specific scenario or candidate. The descriptors "paint a picture" of the types of behaviors likely observed for a given performance domain and a given level (BIN) of performance. The descriptors should allow evaluators to compare what they observed with the general nature of what is described at different levels.

To qualify for a rating within one of the BINS, overall performance must be assessed as most similar to the types of behaviors listed in that BIN's descriptors. Upper-level performances should show frequent and consistent behaviors similar to those described, but there may be occasional lapses to lower levels. Similarly, lower-level performances should show frequent or consistent behaviors similar to those described, but with occasional performance at higher levels. At the middle level, some excursions to upper and lower levels may occur.

To make NTS ratings, the evaluator should:

- Observe the entire scenario performance, perhaps taking notes on performance in the 4 domains
- Choose the BIN ('Poor', 'Average', or 'Excellent') that best describes the overall performance of the individual or team being evaluated
- Then decide on the sub-score within that BIN, determining whether the observed performance was closer to the lower performance belonging to that BIN, in the middle of that bin's performance, or closer to the upper behavior within that BIN. A higher frequency or consistency of behaviors in one direction or another may influence the choice of numerical rating. The occurrence of occasional behavioral outliers outside the bin may also influence the choice.

**
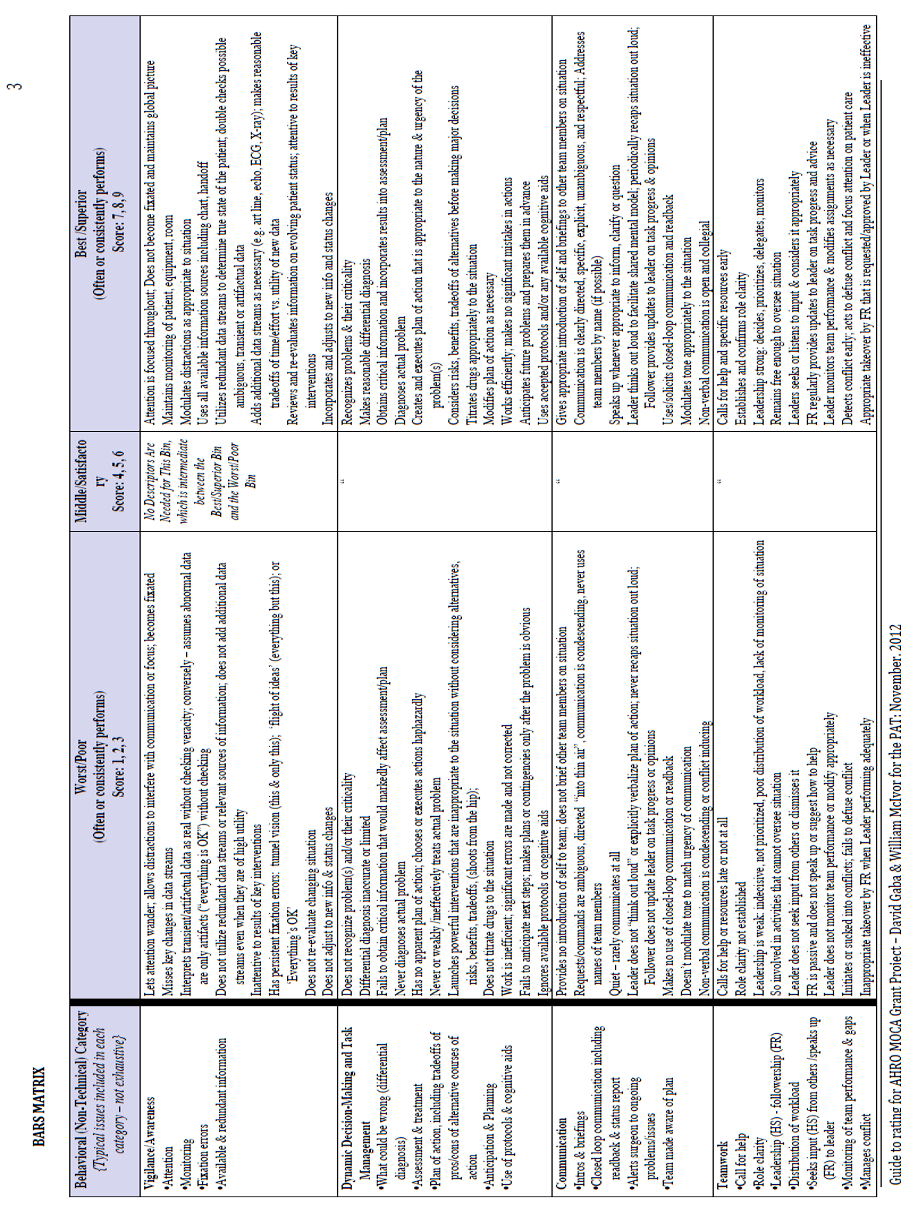
**

Using 'vigilance' as an example, a real-time evaluator might observe a participant initially caught in a fixation error, but then quickly notice another clinical clue and begin to develop a broader differential diagnosis; they might request other data (an arterial blood gas, for example), interpret this information correctly, but end up getting distracted by an ECG artifact. After observing the entire performance, the BNTSRS determines whether the performance was poor or excellent. If it's neither, they determine that the 'vigilance' performance was average. In this example, the BNTSRS might think that the person's 'vigilance' score was closer to excellence, and make the determination that this score should be in the 'Excellent' bin. Now, the BNTSRS considers the degree of excellence (evaluating the amount of time the participant's vigilance was excellent and the degree and magnitude of lapses in 'poor' or 'average' behavior exhibited) and determines whether the participant was closer to the poor-excellent boundary, upper-excellent, or if determined to be neither of these, middle-excellent. In this example, the evaluator determined there were sufficient vigilance lapses for this subject to behave closer to the 'poor-excellent' boundary and therefore assigned them a score of 6 for this non-technical behavior element.

**HOLISTIC AND GLOBAL RATINGS:**
After scoring the individual elements of the HS’ non-technical performance, the RTRs rate the holistic (or overall) medical/technical and non-technical/behavioral performance of the HS **and** the team (HS & FR) for the whole scenario. These ratings use the same 9-point scale and algorithm for determination as described above – first, the RTR considers the HS’ medical/technical performance in-toto for the scenario, and assigns it to a BIN (it was either poor or excellent, or, if neither of those, then it was medium). Then they pick the relevant sub-score for that BIN. After giving a holistic score for technical performance, the RTR uses the same algorithm to determine the holistic non-technical skill rating. The score sheet (see example below) provides places for a numerical score for each of the two holistic questions.

**
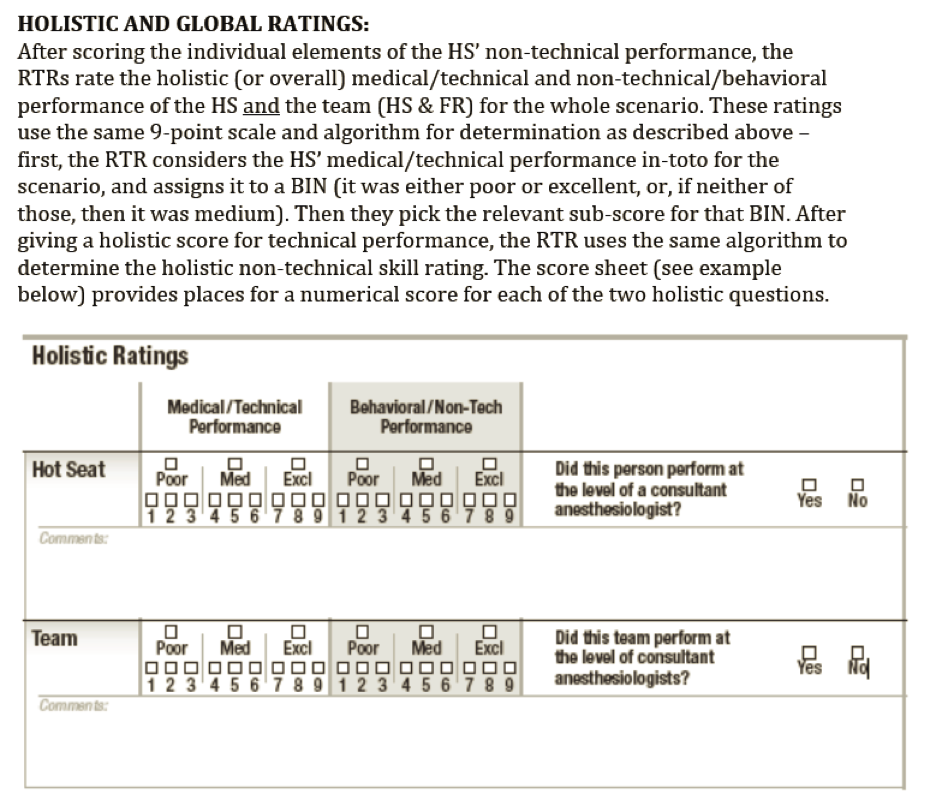
**

**Supplemental Material D - System Usability Scale (SUS) [Brooke J. In: Jordan P, Thomas B, McClelland I, Weerdmeester B, eds. SUS: A 'Quick and Dirty' Usability Scale, Usability Evaluation in Industry. London, UK: Taylor & Francis; 1996:189-194].**

**This 10-item scale assesses system usability, with responses on a 5-point Likert scale from 1 (Strongly Disagree) to 5 (Strongly Agree).**

1. **I think I would like to use this system frequently.**

Strongly Disagree [1] [2] [3] [4] [5] Strongly Agree

1. **I found the system unnecessarily complex.**

Strongly Disagree [1] [2] [3] [4] [5] Strongly Agree

1. **I thought the system was easy to use.**

Strongly Disagree [1] [2] [3] [4] [5] Strongly Agree

1. **I think I would need support of a technical person to be able to use this system.**

Strongly Disagree [1] [2] [3] [4] [5] Strongly Agree

1. **I found the various functions in this system were well integrated.**

Strongly Disagree [1] [2] [3] [4] [5] Strongly Agree

1. **I thought there was too much inconsistency in this system.**

Strongly Disagree [1] [2] [3] [4] [5] Strongly Agree

1. **I would imagine that most people would learn to use this system very quickly.**

Strongly Disagree [1] [2] [3] [4] [5] Strongly Agree

1. **I found the system very cumbersome to use.**

Strongly Disagree [1] [2] [3] [4] [5] Strongly Agree

1. **I felt very confident using the system.**

Strongly Disagree [1] [2] [3] [4] [5] Strongly Agree

1. **I needed to learn a lot of things before I could get going with this system.**

Strongly Disagree [1] [2] [3] [4] [5] Strongly Agree

**Supplemental Material E - ISO 9241-400 Ergonomics Scale.**

**This 6-item instrument evaluates ergonomic aspects of the system interface, with items rated on a 5-point Likert scale from 1 (Strongly Disagree) to 5 (Strongly Agree), in accordance with ISO 9241-400 standards.**

**1. The ML1 device is too bulky or too heavy.**
Strongly Disagree [1] [2] [3] [4] [5] Strongly Agree

**2. The mental effort (concentration) required to operate the device was very high.**Strongly Disagree [1] [2] [3] [4] [5] Strongly y Agree

**3. Arm and hands/fingers fatigue were very high.**
Strongly Disagree [1] [2] [3] [4] [5] Strongly Agree

**4. Eye fatigue was very high.**
Strongly Disagree [1] [2] [3] [4] [5] Strongly Agree

**5. Head fatigue was very high.**
Strongly Disagree [1] [2] [3] [4] [5] Strongly Agree

**6. I would be comfortable using the device for a long time.**
Strongly Disagree [1] [2] [3] [4] [5] Strongly Agree
